# Cerebral Thromboembolism after Lobectomy for Lung Cancer: Pathological Diagnosis and Mechanism of Thrombus Formation

**DOI:** 10.3390/cancers11040488

**Published:** 2019-04-05

**Authors:** Hirotsugu Hashimoto, Genki Usui, Yuta Tsugeno, Keisuke Sugita, Gulanbar Amori, Teppei Morikawa, Kentaro Inamura

**Affiliations:** 1 Department of Diagnostic Pathology, NTT Medical Center Tokyo, Tokyo 141-8625, Japan; gusui-tky@umin.ac.jp (G.U.); tmorikawa-tky@umin.ac.jp (T.M.); 2 Faculty of Healthcare, Tokyo Healthcare University, Tokyo 141-8648, Japan; 3 Division of Pathology, The Cancer Institute, Japanese Foundation for Cancer Research, Tokyo 135-8550, Japan; yuta.tsugeno@jfcr.or.jp (Y.T.); obulhasim.gulanbar@jfcr.or.jp (G.A.); 4 Department of Comprehensive Pathology, Graduate School of Medical and Dental Sciences, Tokyo Medical and Dental University, Tokyo 113-8510, Japan; keisuke.integralugita@gmail.com

**Keywords:** thrombus, pathology, cerebral thromboembolism, lung cancer, lobectomy

## Abstract

Lung cancer is the leading cause of cancer-related deaths worldwide. Although molecular therapies have emerged as efficacious strategies for the treatment of lung cancer, surgical resection is still recommended as a radical therapeutic option. Currently, lobectomy is regarded as the most reliable radical treatment of primary lung cancer. Among the various complications after lobectomy, cerebral thromboembolism requires attention as a life-threatening complication during the early postoperative period. It occurs in 0.2–1.2% of surgical cases of lung cancer and typically develops following left upper lobectomy with a long pulmonary vein stump (PVS). PVS-associated thrombosis is known to cause cerebral thromboembolism after such procedures; however, distinguishing this specific complication from that caused by postoperative atrial fibrillation is challenging. We summarize herein the diagnostic pathology of thrombus formation in accordance with its thrombogenic mechanism. We focus on the potential utility of the pathological assessment of thrombectomy specimens. The morphological information obtained from these specimens enables the presumption of thrombogenic etiology and provides useful clues to both select an appropriate pharmacotherapy and determine a follow-up treatment for cerebral thromboembolism.

## 1. Introduction

Lung cancer is the leading cause of cancer deaths globally [1,2]. Although molecular therapies have emerged as efficacious strategies for the treatment of lung cancer [3,4,5,6], surgical resection is still recommended as a radical therapeutic option. Sublobar resection (segmentectomy or wedge resection) has been recently utilized for cases of small-sized non-small cell lung carcinoma (NSCLC); however, lobectomy remains the most reliable radical treatment of primary lung cancer. Lobectomy requires the amputation of involved pulmonary vessels [7,8]. Various complications after lobectomy, including bleeding, infection, atelectasis, air leak, pneumothorax, chylothorax, empyema, and cardiac complications (e.g., atrial fibrillation (AF) and cardiac tamponade) have been observed [9,10,11,12,13,14]. Among these complications, cerebral thromboembolism is a life-threatening condition during the early postoperative period, occurring in 0.2–1.2% of surgical lung cancer cases [14,15,16,17,18].

Cerebral thromboembolism most commonly develops after left upper lobectomy [16,17,18,19,20] but rarely after left lower lobectomy [16,21]. As left upper lobectomy generally requires a longer pulmonary vein stump (PVS) than other types of lobectomy, the longer PVS is susceptible to thrombogenesis via blood stasis, resulting in cerebral thromboembolism [18,19,20]. Aside from PVS-associated thrombogenesis, paroxysmal AF, which is a common complication after general thoracic surgeries, also causes cerebral thromboembolism. While it is challenging to identify the precise etiology of a thrombus, determining this information is important for the selection of the most appropriate pharmacotherapy and follow-up treatment for patients with chronic cerebral thromboembolism. Some may believe that cerebral thromboembolism after lung cancer surgery is attributable to Trousseau’s syndrome; however, various factors affecting the thrombogenic mechanism must be considered. We herein review the mechanism by which cerebral thromboembolism develops after lobectomy for lung cancer with a special focus on the pathogenesis of thrombus formation and its pathological diagnosis.

## 2. Mechanism of Thrombus Formation (Virchow’s Triad)

### 2.1. Normal Hemostasis and the Coagulation Cascade

**Figure 1:**
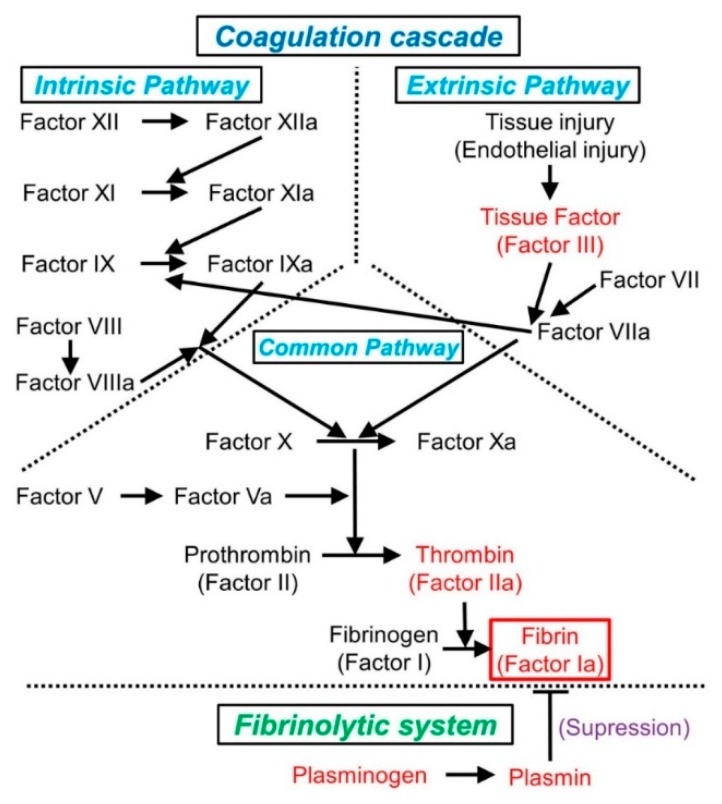
The coagulation pathway and its relationship to the fibrinolytic system (modification of reference [22]). In the coagulation cascade, the activation of factor X is initiated by both extrinsic and intrinsic pathways, which converge in the formation of a fibrin plug, which is degraded by plasmin in the fibrinolytic system. Activated factors are indicated with a lowercase “a”.

To better understand the thrombogenic mechanism, it is necessary to comprehend the general sequence of hemostasis and the coagulation cascade (Figure 1).

In cases of tissue injury, the endothelium is damaged and vascular constriction is regulated by the neurogenic reflex and locally secreted factors (e.g., endothelium-derived endothelin) [22,23]. In the damaged endothelium, both platelet adherence and activation are triggered by the exposure of the highly thrombogenic subendothelial extracellular matrix (ECM). Subsequently, hemostatic plugs, composed of platelet aggregates, develop during primary hemostasis [22]. Thereafter, the transmembrane receptor tissue factor (TF, also known as factor III, an endothelium-derived procoagulant protein) is exposed at the site of the lesion [22]. Through its interaction with factor VII, TF triggers the coagulation cascade, eventually converting prothrombin into thrombin. During secondary hemostasis, thrombin cleaves circulating fibrinogen into insoluble fibrin, generates a network of fibrin mesh, and recruits platelets to the injured site (Figure 1) [22,23]. Solid plugs are formed by polymerized fibrin and platelet aggregates [22].

In the blood coagulation cascade, factor X (the initiator of the common pathway) is activated via two distinct pathways (extrinsic and intrinsic) (Figure 1). The extrinsic pathway is triggered by endothelial damage related to tissue injury, whereas the intrinsic pathway is activated only upon exposure of factor XII to a thrombogenic surface. Although separate, these pathways interconnect at several points [22]. For example, factor IX is activated by factor XIa but is also activated via its interaction with factor VIIa [22] (Figure 1). Both the extrinsic and intrinsic pathways lead to the common pathway, terminating in the formation of fibrin plugs, which are degraded by plasmin in the fibrinolytic system [22,24].

In clinical laboratory testing, the function of the coagulation cascade is typically assessed by prothrombin time (PT) and activated partial thromboplastin time (APTT). The function of the extrinsic pathway (factors VII, X, II, and V, and fibrinogen) is assessed by PT, whereas the function of the intrinsic pathway (factors XII, XI, IX, VIII, X, and V, prothrombin, and fibrinogen) is evaluated by APTT [22].

Recent studies developed the cell-based model of hemostasis and revealed the mechanisms by which cellular elements influence the hemostatic process [25,26]. According to this model, coagulation occurs on the surface of platelets and/or other TF-exposing cells via the following three phases: (i) initiation, which occurs on TF-bearing cells; (ii) amplification, in which platelets and co-factors are activated to trigger large scale thrombin generation on the platelet surface; and (iii) propagation, in which large amounts of thrombin are generated on the surface of activated platelets [25,26]. This cell-based model provides an explanation of hemostasis from diversified views, as opposed to a protein-based model [25,26].

### 2.2. Mechanism of Thrombosis (Virchow’s Triad)

**Figure 2:**
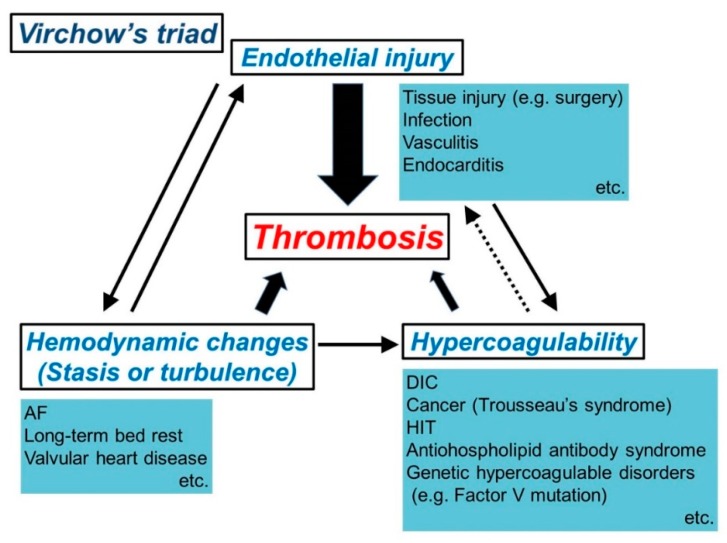
Scheme of thrombosis triggered by Virchow’s triad (modification of reference 22). Thrombosis is caused by endothelial injury, abnormal blood flow (e.g., stasis and turbulence of blood flow), and/or hypercoagulability. These three factors interact with each other. AF, atrial fibrillation; DIC, disseminated intravascular coagulopathy; HIT, heparin-induced thrombocytopenia.

The concept of Virchow’s triad, which involves three factors (i.e., endothelial injury, hemodynamic changes (stasis or turbulence), and hypercoagulability), is used to identify the thrombogenic mechanism under abnormal conditions (Figure 2) [22,24,27].

Among these three factors, endothelial injury is the most influential in terms of cardiac and arterial thromboses, and it is usually accompanied by traumatic or inflammatory injury of the vascular endothelium or endocardium. Endothelial injury causes exposure of the subendothelial ECM, leading to platelet adherence and activation. Furthermore, dysfunctional endothelial cells can generate high levels of procoagulant factors (e.g., platelet adhesion molecules, TF, and plasminogen activator inhibitors) and may produce fewer anticoagulant effectors (e.g., thrombomodulin and prostacyclin) [22].

Hemodynamic changes (stasis or turbulence) can increase procoagulant activity and leukocytic adhesion by modulating endothelial cell gene expression [22]. Hemodynamic changes also inhibit washout or dilution of clotting activators via the circulation of fresh blood [22]. While blood stasis is a major cause of venous thrombosis [28], turbulent blood flow can contribute to cardiac and arterial thromboses. Notably, turbulent blood blow can also induce thrombogenesis by injuring the endothelium, suggesting a close interaction between hemodynamic changes and endothelial injury (Figure 2).

Hypercoagulability is also important in thrombosis because clotting factors themselves trigger thrombogenesis and enhance thrombosis in genetic hypercoagulable states (e.g., mutations in factor V and prothrombin) [22]. Furthermore, thrombosis due to other hypercoagulable conditions (e.g., disseminated intravascular coagulopathy (DIC), heparin-induced thrombocytopenia, and Trousseau’s syndrome) can also be attributed to hypercoagulability.

Virchow’s triad is conceptually composed of the above-mentioned three factors, which are closely associated with each other during thrombogenesis under abnormal conditions.

### 2.3. Recent Molecular Studies on Thrombosis

Here, we describe recent molecular studies on thrombus formation, specifically those focusing on its associations with platelet-related molecules, neutrophils, and microRNAs (miRNAs).

In thrombogenesis, platelets adhere to ECM largely through the interaction between von Willebrand factor and glycoprotein Ib [22]. Platelet glycoprotein Ib, which acts as a platelet surface receptor in thrombogenesis, has also been associated with the metastatic potential of lung cancer [29]. Granules (particularly α-granules), which are released from platelets just after the adhesion to ECM, contain platelet-derived growth factors [22,30,31]. This type of growth factor has been shown to promote lung carcinogenesis [30,31]. These lines of evidence indicate that platelets play an important role in thrombogenesis as well as in tumorigenesis.

Recent studies have also revealed an important role of neutrophils in thrombogenesis [32,33,34,35]. Neutrophils produce TF in inflammatory conditions, and consequently activate the extrinsic pathway [32]. Furthermore, neutrophils promote thrombogenesis by interacting with platelets and forming neutrophil extracellular traps, which are network structures comprising DNA, histone, and granular antimicrobial proteins (e.g., neutrophil elastase). Through these mechanisms, neutrophils act as important mediators in thrombogenesis [32,33,34,35].

While miRNAs have been associated with cancer development, in the field of cardiovascular pathology, their association with atherosclerosis has also been investigated in detail [24]. Hemodynamic shear stress has been associated with miRNAs that modulate endothelial proliferation and inflammation [36,37]. For instance, miRNA-92a inhibits the transcription of Krüppel-like factor 2 (KLF2) and KLF4. These KLF genes, known as atheroprotective genes, suppress thrombosis by regulating the differentiation of smooth muscle cells in the vascular wall [38,39]. Both KLF2 and KLF4 have been shown to act as tumor suppressors in NSCLC, attracting attention in the field of thoracic oncology [40,41,42].

A number of recent studies have investigated the processes by which cell-to-cell transport occurs via miRNAs within extracellular vesicles or exosomes [43,44]. Additional effort should be focused on understanding the molecular pathophysiology of the thrombogenic process for the better management of thromboembolism.

## 3. Trousseau’s Syndrome

### 3.1. Overview

Trousseau’s syndrome is defined as a coagulopathology associated with visceral cancer and is considered a paraneoplastic syndrome [36]. It was initially considered a condition of cancer-associated thrombophlebitis, especially deep vein thrombosis (DVT) and pulmonary thromboembolism (PTE), leading to the discovery of an underlying cancer. Armand Trousseau, a French neurologist, recognized this syndrome and reported three cases of cancer-associated DVT in the 1860s [27,45,46]. He himself suffered from a similar condition, resulting from gastric cancer [45]. Recently, the concept of Trousseau’s syndrome has been expanded to include venous and arterial cancer-associated coagulopathies (e.g., microangiopathy, verrucous endocarditis, and arterial emboli caused by DIC) [27,45,47].

Although Trousseau’s syndrome is widely recognized by physicians, its exact definition has not yet been established. Some insist that cancer-associated thrombosis accompanied by a hypercoagulopathy is included in the definition of Trousseau’s syndrome. On the other hand, some physicians strictly use this term only in cases of cancer-associated venous thrombosis that lead to the discovery of an underlying cancer [48,49]. In this review, we use the term “Trousseau’s syndrome” for both venous and arterial cancer-associated coagulopathies, but do not include a hypercoagulopathy accompanied by a noncancer-associated coagulopathy in the broader definition of Trousseau’s syndrome, in line with a previous review of this condition [45].

### 3.2. Clinical Characteristics Related to Cerebral Thromboembolism

Trousseau’s syndrome has been used to describe various conditions, ranging from its original definition (a state of cancer-associated thrombophlebitis, especially DVT and PTE) to any kind of cancer-associated coagulopathy [27,45,48,49]. A prior study of autopsy cases reported that about 7.5% of cancer patients suffer a cerebral infarction during their illness [50]. Among these cases, nearly one-half were attributed to nonbacterial thrombotic endocarditis (NBTE) or DIC [50], which resulted from hypercoagulable states that were attributable, at least in part, to sialomucin produced by an adenocarcinoma [51,52]. Indeed, adenocarcinoma, especially originating from the digestive, gynecological, and respiratory organs, is the leading histological type of cancer-associated with Trousseau’s syndrome [27,45].

### 3.3. Pathogenesis

Trousseau’s syndrome is not explainable by a simple mechanism. Numerous definitions of this condition appear to result from diverse pathophysiological mechanisms underlying cancer-associated hypercoagulable states.

As noted earlier, a cancer-derived mucin has been suggested to trigger Trousseau’s syndrome [51,52]. This potential correlation may explain the higher rate of adenocarcinoma compared to other histological types associated with this syndrome. Sialic acid, a component of mucin, activates TF, initiating fluid-phase blood coagulation [53,54]. Tumor-derived gangliosides, which are a major source of sialic acid, are also known to enhance platelet adhesion to ECM at the initial step of platelet activation [55]. However, the hypercoagulable state in cancer patients cannot be explained only by mucin, because not all cases of Trousseau’s syndrome are associated with a mucin-secreting cancer [47]. Various cancers contain TF at high concentrations; therefore, cancer-derived TF itself is also associated with Trousseau’s syndrome [54]. Via its interactions with TF, factor VII is converted into its activated form (i.e., factor VIIa) in the extrinsic pathway of the coagulation cascade (Figure 1). Factor VIIa activates factor X to produce thrombin, which is necessary for platelet activation and fibrin production [45].

In proliferating cancer cells, tumor hypoxia upregulates the expression of coagulation-promoting genes (e.g., TF and plasminogen activator inhibitor type 1) and causes a hypercoagulable state [56]. Furthermore, experimental evidence demonstrates that an activated MET oncogene—known as a key regulator of the resistance of NSCLC to gefitinib (an EGFR inhibitor)—promotes cancer-associated thrombosis [57,58].

Taken together, Trousseau’s syndrome is well recognized as thrombotic disorders that are not only due to cancer-derived mucin or TF, but also to other thrombogenic mechanisms, such as tumor hypoxia and MET gene activation [45].

### 3.4. Association with Cerebral Thromboembolism after Lung Cancer Surgery

Is cerebral embolism after lung cancer surgery attributable solely to Trousseau’s syndrome? Indeed, the most common histological type of lung cancer in cases of cerebral thromboembolism after lobectomy is adenocarcinoma, similar to Trousseau’s syndrome [18]. However, cerebral thromboembolism after lung cancer surgery cannot be fully explained by Trousseau’s syndrome. First, blood stasis due to a long PVS causes thrombosis in 3.3–3.6% cases of lobectomy [18,19]. Second, the removal of a pulmonary lobe that does not contain lung cancer can also lead to cerebral embolism [59], suggesting that lobectomy itself is a risk factor for cerebral embolism. Additionally, paroxysmal AF, a common complication after general thoracic surgeries, can cause cerebral embolism [14]. Taken together, we strongly suggest that cerebral thromboembolism after lung cancer surgery be excluded from this syndrome.

To identify the pathological characteristics of thrombus that cause cerebral thromboembolism in Trousseau’s syndrome, a previous study examined patients with stage IV cancer (including lung and pancreas cancers) and DIC [60], which reflect a hypercoagulable state. This study showed that thrombi in patients with this syndrome contained extreme amounts of fibrin [60]. Understanding thrombus pathology will help determine the degree to which cancer-associated hypercoagulability can be attributed to cerebral thromboembolism after lung cancer surgery.

## 4. Mechanism of Cerebral Thromboembolism after Lobectomy for Lung Cancer

### 4.1. Overview

Cerebral thromboembolism is a rare, life-threatening complication after lung cancer surgery, particularly after pulmonary lobectomy. This complication occurs in 0.2–1.2% of cases of lung cancer surgery [14,15,16,17,18].

**Figure 3:**
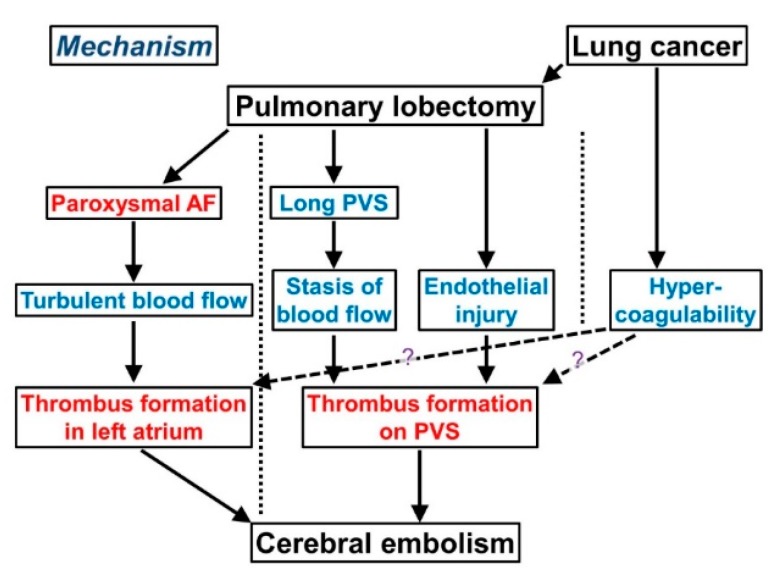
Mechanism of cerebral embolism after lobectomy for lung cancer. Thrombi are formed via two distinct pathways: atrial fibrillation (AF) and pulmonary vein stump (PVS). Hypercoagulability induced by lung cancer may induce thrombus formation via these two pathways.

We summarize the mechanism by which cerebral thromboembolism develops after lobectomy (Figure 3), referring to the general mechanism of thrombosis (Virchow’s triad) described in Section 2 (Figure 2). Atherogenicity may cause a cerebral embolism immediately after lung cancer surgery, but we do not discuss atherogenicity-associated cerebral embolisms here.

### 4.2. Pulmonary Vein Stump (PVS)

A PVS can be a thrombogenic site of cerebral thromboembolism after lobectomy because the pulmonary vein, unlike the other veins, directly connects to the left cardiac system. This anatomical feature explains the relatively high frequency of cerebral thromboembolism in the PVS. Whereas pulmonary lobectomy can generate a thrombus in the PVS, only a small portion of the thrombus eventually leads to a cerebral thrombosis [18,19,20]. Such a thrombus in the PVS usually develops within three months after surgery [19]; conversely, one year or more after lobectomy, a thrombus rarely develops in the PVS [61,62].

The length of the PVS is an important factor affecting thrombus formation after lobectomy. Some have suggested that the length of the PVS should be short to prevent a lobectomy-induced cerebral embolism; however, a short PVS in lobectomy, which requires the amputation of the pulmonary vein near the pericardium, can cause cardiac tamponade, which is a more critical complication than cerebral embolism [12,13]. Thus, an appropriate length for the PVS must be determined to avoid complications after lobectomy.

As with blood stasis, endothelial injury also plays an important role in thrombogenesis in the PVS. Pulmonary vein amputation during lobectomy injures the endothelium and consequently activates the extrinsic pathway of the coagulation cascade (Figure 1). Cerebral thromboembolism develops in approximately 3% of patients under anticoagulation therapy after left atrial appendage occlusion [63,64], although the identification of the source of the embolism is challenging [65]. Because the left atrium connects to the systemic circulation, cardiac surgery can potentially lead to cerebral thromboembolism via the development of NBTE due to endothelial injury. Lung surgery during transplantation can also cause cerebral thromboembolism via thrombus formation in the pulmonary vein [59]. These observations suggest an association between endothelial injury and thrombogenesis in the pulmonary vein without blood stasis.

Taken together, both blood stasis and endothelial injury can result in thrombus formation in the PVS after lobectomy (Figure 3).

### 4.3. Atrial Fibrillation (AF)

AF increases the risk of cerebral thromboembolism by the formation of a thrombus in the atrium due to turbulent blood flow (Figure 3). AF, particularly paroxysmal AF, is a common complication in approximately 14–20% of cases of lung surgery [14,66]. AF-associated thromboembolism is thought to result from turbulent blood flow due to AF in the left atrium, especially in the left atrial appendage. Electrophysiological observations suggest that the majority of AF cases arise from the pulmonary vein [67]. A myocardial sleeve (an extension of myocardial tissue from the left atrium) is observed in 80–100% of all pulmonary veins [68,69,70]; catheter ablation of the sleeved tissue is an established treatment of AF [71,72]. Recent studies reported that left atrial appendage occlusion decreases the risk of thromboembolism and improves survival in AF patients undergoing cardiac surgery [63,64]. These findings indicate that AF causes cerebral thromboembolism via thrombus formation in the left atrial appendage.

Taken together, AF after lung surgery, including lobectomy for lung cancer, is a risk factor of cerebral thromboembolism via thrombus formation in the left atrial appendage (Figure 3). The pathogenesis of thrombus during AF differs from that in the PVS. It should be noted that AF-associated thrombus is attributable to the turbulence of blood flow, whereas PVS-associated thrombus results from blood stasis and endothelial injury.

### 4.4. Possibility of Cancer-Associated Hypercoagulability

As described in Section 3 (Trousseau’s syndrome), we do not define cerebral thromboembolism after lobectomy only due to a cancer-associated hypercoagulability as Trousseau’s syndrome. However, thrombogenesis after lobectomy may share a similar mechanism with that of Trousseau’s syndrome in terms of a cancer-associated hypercoagulability.

As previously described, the removal of a pulmonary lobe that does not contain a neoplasm carries a risk of cerebral embolism [59], suggesting its causal association with lobectomy. Moreover, patients with early stage cancer (stage I or stage II) more frequently suffer a cerebral thromboembolism after lobectomy than those with advanced cancer [18,20]. This fact suggests that the major cause of cerebral thromboembolism is not cancer, but lobectomy itself. Taken together, a cancer-associated hypercoagulability is only partially attributable to cerebral thromboembolism after lobectomy for lung cancer. 

## 5. Thrombectomy and Thrombus Pathology in Practice

### 5.1. Development of Thrombectomy

Thrombectomy has recently developed as an effective treatment for cerebral ischemic stroke [73,74,75], although the hyperacute phase is generally treated by pharmacotherapeutic recanalization, such as with tissue plasminogen activator. Meanwhile, recent studies recommended an expanded application of thrombectomy, according to both radiologic imaging (particularly, magnetic resonance imaging) and duration from onset [76,77]. Due to advances in thrombectomy and increased numbers of removed thrombus specimens, the importance of thrombus pathology has increased. Although it is important to discern whether the ischemic stroke is cardiogenic for the proper application of pharmacotherapy, its etiology is unclear in nearly 40% of cases despite substantial clinical effort. Such cases of unknown etiology are classified as cryptogenic ischemic stroke [78]. Several studies have described the morphological differences between cardiogenic and noncardiogenic thromboemboli [79,80,81], suggesting the utility of thrombus pathology. We describe thrombus pathology in terms of thrombogenesis.

### 5.2. General Principles of Thrombus Pathology

The trigger for thrombus formation can be determined by pathological characteristics, based on each thrombogenic mechanism (Figure 1 and Figure 2). We list the general principles of thrombus pathology that help determine the etiology of the thrombus as follows:

(1). A thrombus formed by endothelial injury is characterized by an increased number of neutrophils. Endothelial injury also activates the extrinsic pathway of the coagulation cascade, resulting in higher levels of fibrin in the thrombus.

(2). A thrombus formed by blood flow stasis, especially a venous thrombus, is characterized by abundant erythrocytes [22].

(3). A thrombus triggered by turbulent blood flow does not contain abundant erythrocytes but can manifest abundant neutrophils with a co-existing endothelial injury.

(4). A hypercoagulability results in the formation of a thrombus with a dense fibrinous material.

(5). A thrombus that develops at a distant site (e.g., PTE secondary to DVT, cerebral thromboembolism secondary to AF, and cerebral embolism secondary to the rupture of a carotid arterial atheroma) is composed of two components, one formed distantly from the occlusion site and the other formed via blood stasis at the occlusion site. As these two components are usually admixed, it is challenging to differentiate them, particularly in a thrombus formed by a similar mechanism (i.e., blood stasis in PTE secondary to DVT).

From these points of view, we next present examples of typical thrombus pathologies that help identify the etiology of the thrombus.

### 5.3. Examples of Thrombus Pathology

#### 5.3.1. Thromboembolism Due to Disseminated Intravascular Coagulopathy (DIC)

**Figure 4:**
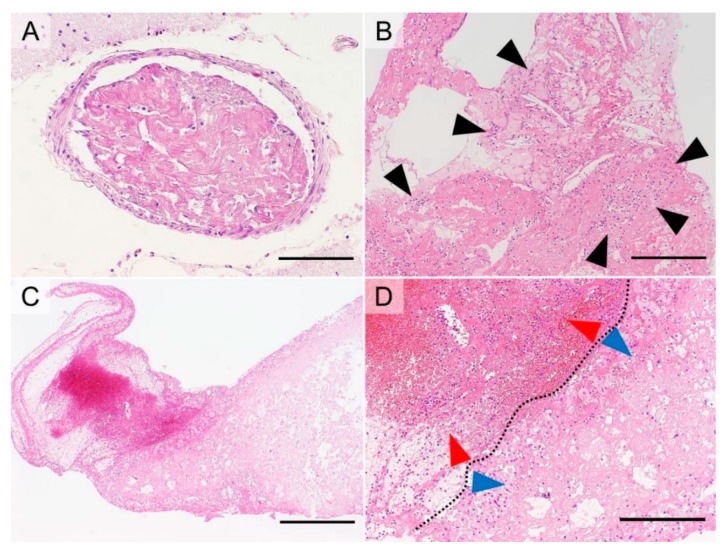
Examples of thrombus morphology (hematoxylin and eosin staining). A thrombus due to disseminated intravascular coagulopathy is composed of dense fibrin (**A**, bar = 125 µm). A coronary atherogenic thrombus contains many atheromas with fibrin deposits accompanied by abundant neutrophils (black arrowheads) (**B**, bar = 250 µm). A cerebral atherogenic embolic thrombus contains two components (**C**, bar = 625 µm; **D**, bar = 125 µm, separated by a dotted line): an erythrocyte-rich component (**D**, red arrowheads) and an atheroma including foamy macrophages (**D**, blue arrowheads).

This type of thrombus develops via hypercoagulability and is characterized by a dense fibrinous material (Figure 4A). A thrombus accompanied by Trousseau’s syndrome often develops via this mechanism [60].

#### 5.3.2. Coronary Atherogenic Thrombus

This type of thrombus results from endothelial injury by the rupture of vulnerable plaques in the coronary artery and is subsequently modified by an activated coagulation cascade due to tissue injury. Because of these thrombogenic processes, coronary atherogenic thrombi contain atheromas with fibrin deposits and abundant neutrophils (Figure 4B).

#### 5.3.3. Atherogenic Thrombus in Cerebral Thromboembolism

This type of thrombus is initially formed by a fragmented atheroma from a ruptured plaque (usually present in the internal carotid artery or aorta) and secondarily modified by blood stasis. Therefore, the thrombus is composed of two components: an atherogenic component and an erythrocyte-rich component resulting from blood stasis (Figure 4C,D).

#### 5.3.4. Thrombus Due to Thrombophlebitis

**Figure 5:**
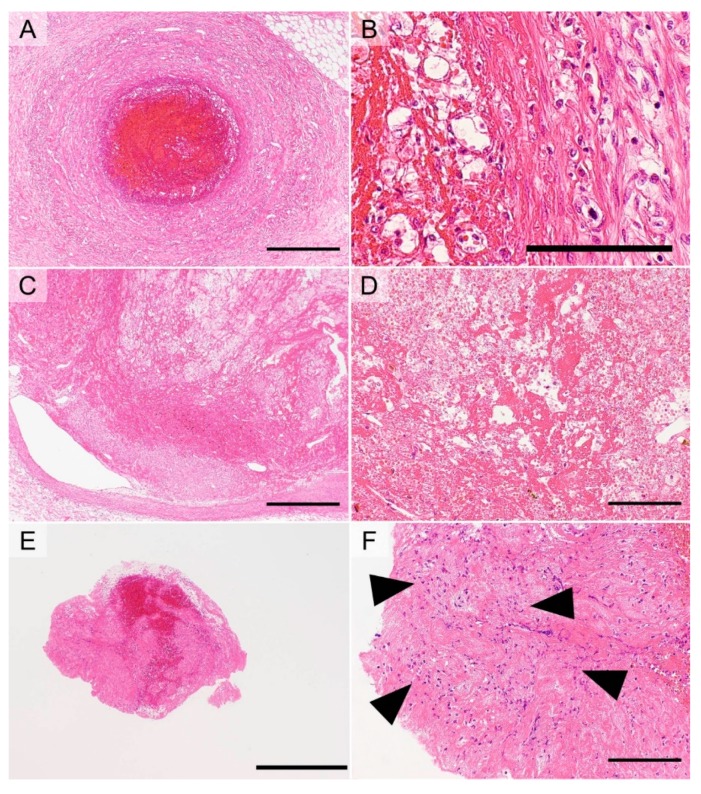
Examples of thrombus morphology (hematoxylin and eosin staining). A thrombus due to thrombophlebitis (**A** and **B**), which contains an increased number of erythrocytes, accompanied by a relatively thick fibrinous material (**A**, bar = 625 µm) and an increased number of neutrophils that infiltrate the venous walls (**B**, bar = 125 µm). A thrombus that caused a pulmonary thromboembolism resulting from deep vein thrombosis (**C** and **D**). This type of thrombus is characterized by abundant erythrocytes and mesh-like fibrin (**C**, bar = 625 µm; **D**, bar = 125 µm). A cardiogenic thrombus due to atrial fibrillation is composed of two components (**E**, bar = 625 µm): a relatively dense fibrinous material, scattered neutrophils (black arrowheads), and a small number of erythrocytes (**F**, bar = 125 µm); and abundant erythrocytes admixed with the former component (**E**).

Thrombophlebitis can result in thrombus formation via both blood stasis and endothelial injury; therefore, this type of thrombus usually contains an increased number of erythrocytes and neutrophils, accompanied by a relatively thick laminar fibrinous material (Figure 5A,B).

#### 5.3.5. Thrombus Due to PTE Resulting from DVT

This type of thrombus develops via blood stasis and is characterized by abundant erythrocytes and a fibrin mesh (Figure 5C,D). The thrombus primarily develops at a distant site, such as in a lower limb, and travels from the primary site to the lung, where the thrombus can cause an occlusion and subsequently undergo modification via blood stasis. Therefore, the thrombus may contain two different components; however, it is often challenging to differentiate these two components, because both are formed via blood stasis.

#### 5.3.6. AF-Associated Cardiogenic Thrombus in Cerebral Thromboembolism

This type of thrombus develops by turbulent blood flow as well as by endothelial injury within the left atrium, where blood flow is rapid. The thrombus then reaches and occludes the cerebral artery where the thrombus is modulated by blood stasis. Because of these processes, the thrombus is composed of two components. One component, which results from turbulent blood flow and endothelial injury, contains a relatively dense fibrinous material, scattered neutrophils, and a small number of erythrocytes (Figure 5E,F) [79,80,81]. The other component, which is secondarily formed by blood stasis in the occluded cerebral artery, contains abundant erythrocytes (Figure 5E). These two components are usually admixed (Figure 5E).

### 5.4. Utility of Thrombus Pathology for Pharmacotherapy

Most mechanisms underlying thrombogenesis can be determined based on the morphological characteristics of the thrombus, although some may be unexplainable based on only morphological assessments. In clinical practice, the etiology of the thrombus is based on its morphological characteristics. In such situations, clinical information (e.g., the existence of AF or vulnerable plaques in the internal carotid artery) should also be considered. The integration of clinical information and the morphological characteristics is required to determine the etiology of the thrombus.

To choose the most appropriate pharmacotherapy for cerebral embolism, the thrombogenic etiology must first be determined. For example, anticoagulation therapy should be applied for cardiogenic thrombosis, whereas antiplatelet therapy is applicable for atherogenic embolism. Pathological assessments of the thrombus are beneficial to determine the most appropriate pharmacotherapy for cerebral thromboembolism [80]; therefore, much effort should be given to identify the etiology of the thrombus.

## 6. Use of Thrombus Pathology to Treat Post-Lobectomy Stroke

**Figure 6:**
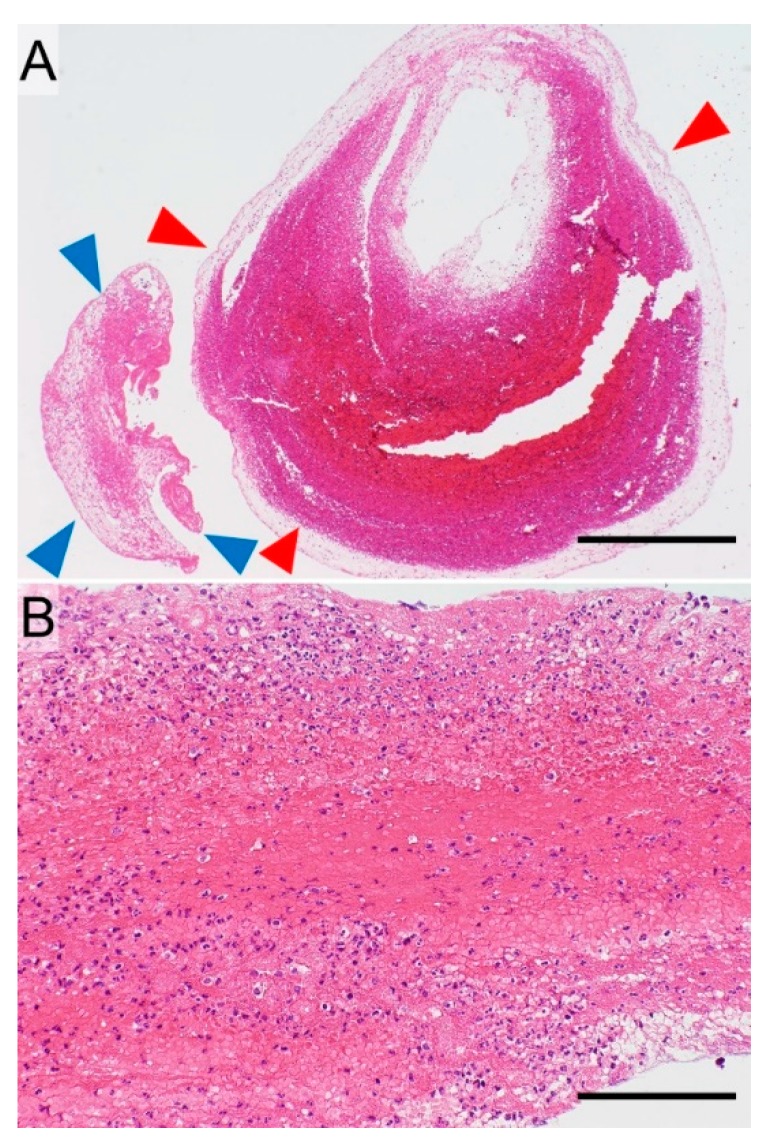
Morphology of a cerebral thromboembolus formed within the pulmonary vein stump (PVS) after pulmonary lobectomy for lung cancer (hematoxylin and eosin staining). This type of thrombus is composed of two components (**A**, bar = 625 µm): a laminar fibrinous component (blue arrowheads) and an erythrocyte-rich component accompanied by a thin mesh-like fibrin network (red arrowheads). The former component is composed of laminar fibrin along with abundant neutrophils (mimicking purulent exudate) and abundant erythrocytes (**B**, bar = 125 µm).

As noted in Section 4, there are two major etiologies that cause cerebral thromboembolism after pulmonary lobectomy for lung cancer (Figure 3): formation in the left atrium due to paroxysmal AF and formation in the PVS due to blood stasis and endothelial injury. While difficult to distinguish, knowing the etiology is required to select the most appropriate pharmacotherapy and follow-up treatment since different etiologies of thrombus formation have distinct pathological features. When the thrombus is modified via blood stasis in the occluded cerebral artery, its composition changes (Figure 6A). The component primarily formed in the PVS is composed of laminar fibrin, abundant neutrophils (reflecting endothelial injury), and erythrocytes (reflecting blood stasis) (Figure 6B) [21], whereas the component in the occluded cerebral artery contains many erythrocytes accompanied by a thin, mesh-like fibrin network (reflecting blood stasis) (Figure 6A).

The major difference between AF- and PVS-associated thrombi is the quantity of the neutrophils and erythrocytes. However, the secondary component of the thrombus in the cerebral artery makes differentiation between them difficult. Taken together, while thrombogenic etiology can be presumed by morphological assessments of the thrombus, the identification of thrombus etiology is not easy.

If the cerebral embolism is caused by paroxysmal AF after pulmonary lobectomy, the patient should be treated with anticoagulation therapy in reference to the risk stratification schemes, such as CHADS2, CHA2DS2-VASc, and HAS-BLED [72,82,83]. On the other hand, the optimal pharmacotherapy for PVS-associated cerebral thromboembolism remains unknown. However, the thrombus pathology may suggest the thrombogenic mechanism and lead to the appropriate choice of pharmacotherapy. For example, we presume that patients with a PVS-associated cerebral embolism should be treated with an anticoagulant, but not with antiplatelet therapy, because PVS-associated thrombus formation is associated with blood congestion [18,19,20], which is supported by histological findings of an erythrocyte-rich thrombus. A case study reported that a patient who was treated with antiplatelet drugs suffered recurrent PVS-associated thrombosis for more than one year after pulmonary lobectomy for lung cancer [62], suggesting the need for anticoagulation therapy in this case. More efforts are needed to establish an effective pharmacotherapy for the chronic phase of PVS-associated thrombosis after lobectomy. It should also be noted that patients with PVS-associated cerebral thromboembolism require follow-up screening for recurrence using contrast-enhanced chest computed tomography.

Thrombectomy is a recently established treatment for thrombosis that enables the collection of thrombus specimens. The thrombogenic etiology can be presumed from the pathological assessment of these specimens and provides useful information for the selection of both optimal pharmacotherapy and follow-up treatment for cerebral thrombosis.

## 7. Conclusions and Future Directions

Cerebral thromboembolism is a critical complication of lobectomy for the treatment of lung cancer. Nonetheless, little information is available about its etiology. Currently, optimal pharmacotherapies and preventive approaches remain unestablished. The relevance of cancer-related hypercoagulability to thrombogenesis in this complication is also unclear. Thrombus pathology has the potential to provide valuable information on the thromboembolic origin, clarify the thrombogenic mechanism, and choose the optimal pharmacotherapy and treatment methods appropriately. As the purpose of cancer surgery is to prolong overall patient survival, much more attention should be given to prevent and treat such a severe complication. Future research in this area by oncologists and cardiovascular pathologists is thus warranted.

## Abbreviations

AF	atrial fibrillation
APTT	activated partial thromboplastin time
DIC	disseminated intravascular coagulopathy
DVT	deep vein thrombosis
ECM	extracellular matrix
KLF	Krüppel-like factor
miRNA	microRNA
NBTE	nonbacterial thrombotic endocarditis
NSCLC	non-small cell lung carcinoma
PT	prothrombin time
PTE	pulmonary thromboembolism
PVS	pulmonary vein stump
TF	tissue factor
